# Effect of Placing Self-Compacting Concrete Bottom up on the Quality of Bond Conditions

**DOI:** 10.3390/ma13102346

**Published:** 2020-05-20

**Authors:** Milena Kucharska, Piotr Dybeł

**Affiliations:** Department of Geomechanics, Civil Engineering and Geotechnics, AGH University of Science and Technology, 30-059 Cracow, Poland; kucharska@agh.edu.pl

**Keywords:** self-compacting concrete, bottom-up placing, pull-out test, bond properties

## Abstract

A self-compacting concrete (SCC) mixture, due to its special rheological properties, may be placed differently than in traditional variants. The paper presents the results of a study on the effect of a bottom-up placing direction on the quality of bond conditions between steel and self-compacting concrete. Elements with overall dimensions of 160 × 480 × 1600 mm consisting of elementary samples with dimensions of the bond test basic modules were constructed. Ribbed steel rebars with diameters of 16 mm were used and located in formworks perpendicularly to the concrete placing direction. Bond tests were carried out according to the pull-out method. The bottom-up placing contributed to the uniformity of the bond conditions quality in the test elements and an overall improvement of the bond properties, especially in its top part. Given the increasing implementation of alternative methods of mix placing and the promising results, the topic should be further investigated.

## 1. Introduction

The unique rheological properties of self-compacting concrete (SCC) are widely known to provide a more effective way of placing it in the form. No additional mechanical compaction is needed as it is properly compacted (with lack of segregation) and vented only under its own weight even in the presence of dense reinforcement.

**Figure 1:**
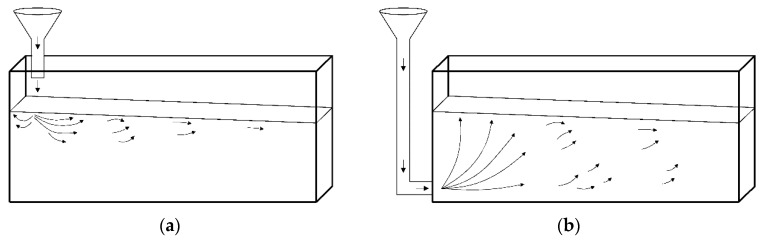
Typical methods of placing self-compacting concrete (SCC): (**a**) pumping from the above, (**b**) pumping from the bottom.

The application of self-compacting concretes in the construction industry brings a number of benefits throughout the entire life cycle of the structure in technological, economic, environmental and social terms. It is associated with the elimination of mechanical compaction, reduction of concrete surface trowelling, hence reduction of concreting and finishing time, material modifications of the mix (replacement of part of cement with mineral additives) and better mechanical characteristics of the hardened concrete. Furthermore, it is possible to manufacture the elements in different variants of concrete mix placing. First of all, it is possible to build an entire beam and wall elements from a single casting point. In the case of many points, it is necessary to adjust their quantity and location to the rheological properties of the SCC mix used, especially its viscosity (wall). Another technological innovation is the possibility of concreting both traditionally from the top (Figure 1a) and the bottom of the form. A technologically appropriate and implemented variant of concreting from below is an injection of the mixture through an inlet connection in the formwork system with a shut-off valve (Figure 1b). According to the general European guidelines for SCC concreting [1], when placing the mix from the bottom, the pumping point should be as close as possible to the middle of the span of the wall, in order to reduce the flow distance.

The execution of elements from the bottom of the form is typically carried out by pumping the concrete mixture, and thus the literature [2,3] presents the advantages and disadvantages of this technology. The benefits of such an approach are primarily the more efficient self-deaeration of the mixture, and consequently the improved surface finish of the element. However, as the pumping is done through a specially prepared valve, there is a local deterioration of the surface finish around it. Additionally, this dedicated shut-off valve complicates the formwork and, therefore, increases its cost-efficiency. Nevertheless, given the fact that the bottom-up technology requires a slightly higher pumping speed with continuous work of the pump, the time of the element completion is shorter and the element itself is devoid of any discontinuities and weak interfaces. In general, the risk of mixture segregation is lower due to the continuous and uniform rise of the mixture in the form, which results in increased strength of concrete. However, in the case of elements with large spans, it is possible that dynamic segregation may occur due to the long flow distance. The main problem and hazard of this technology are the increased formwork pressure [4,5,6]. Thus, it demands additional reinforcement of the formwork and complicates it.

One of the most important aspects of the bond phenomenon is the occurrence of deterioration in the bond conditions along the height of the element i.e., the ‘top bar effect’. This relates to a specific form of segregation that involves the movement of free water in the mixture towards the upper surface of the element, causing the top sections of weaker concrete of higher porosity [7]. At the same time, the quality of the steel-concrete contact is lowered as a result of the settlement of the fresh concrete mixture below the rebar. The effect has been taken into consideration in the international standards [8,9,10], and the decrease in bond strength is compensated for with an appropriate extension of the anchorage length. Despite a relatively comprehensive characterisation of this issue in the literature concerning SCC [11,12,13,14,15,16], in which many factors such as concrete strength, rheological properties of a mixture, length of the bond section etc. have been considered, so far no conclusive results have been obtained regarding the bond phenomenon in SCC. In general, the top bar effect is less significant in SCC concretes than in normal concretes, especially for smaller bar diameters and lower concrete strengths [17,18]. This trend is likely to be caused by the reduced vulnerability of SCC mixtures to bleeding which is associated with increased filler content and the low water-to-binder ratio [19]. For instance, in a study conducted on 1500 mm high columns, [12] made of normal and self-compacting concretes with a varying water-to-cement ratio, the reduction of ultimate bond stress for self-compacting concretes ranged between 32%–55% and from 60% to 74% for normal concretes. However, it has been noted [20] that modification of self-compacting concrete with silica fume in an adequate amount—less than 10%—in relation to the cement mass practically eliminates bond reduction along the height.

The normative guidelines consider this effect via the extension of the anchorage length of rebars in ‘other than good’ bond conditions. However, only the top-down direction of the mix placing is taken into account. Moreover, the binding guidelines are based on the results of tests performed on normal concretes. In the American standard ACI 318 [8], this is an enlargement by 30% of the anchorage length of horizontal rebars with a concrete layer of more than 300 mm underneath. Eurocode 2 [10] is more restrictive in this regard and mentions an extension of the anchorage length of rebars by about 43% located in other than good bond conditions. Furthermore, longer anchorage lengths are required for the bars situated in sliding formworks, which are commonly used in contemporary structures, unless it could be indicated that they belong to an area of ‘good’ bond conditions.

The principal aim of this experimental study is to evaluate the effect of the bottom-up concreting direction on selected bond properties between steel reinforcement and self-compacting concrete. Hitherto, there have been no in-depth studies concerning this effect. However, the technological findings from the implementation on construction sites and laboratory tests (increased pressure on the formwork) and the recommendations for constructing concrete elements in this technology are included in international standards and scientific papers [1,2,21,22,23,24,25]. It is observed that there is a growing interest in this technology within the construction industry, so it would seem appropriate to investigate the effect and its impact on the parameters of the hardened concrete, including its bond to reinforcing bars. Authors have already started working on the project concerning high-perfomance self-compacting concrete (HPSCC) [26]. The authors found that the casting direction has an influence on the phenomenon of the steel–concrete bond in the case of both HPSCC mixtures containing a filler in the form of silica fume and those based entirely on cement. A significant positive impact of bottom-up placing on the uniformity of bond conditions across the height of the elements was identified. The promising results of those studies are encouraging for more comprehensive research. The paper intends to continue the research and observe the phenomenon for self-compacting concrete of normal strength with a filler in the form of fly ash, which is the most commonly used type of SCC in concrete constructions.

## 2. Materials and Methods

### 2.1. Materials

For research purposes, one concrete mix was adopted, the composition of which is presented in Table 1. The mix is based on Portland fly ash cement (CEM II/B-V 32.5R) and fly ash (FA) as a mineral additive. The investigations assumed development of a self-compacting mixture of possibly low strength. The recipe was developed based on common literature and previous experience of the authors.

The investigations were performed on elements with embedded deformed reinforcing bars of 16 mm diameter that were made with reinforcing steel of B500SP class. The rebars met the requirements of EN 10080 [27]. The mean values of the mechanical parameters of the rebars are given in Table 2.

### 2.2. Test Specimens and Basic Modules

**Figure 2:**
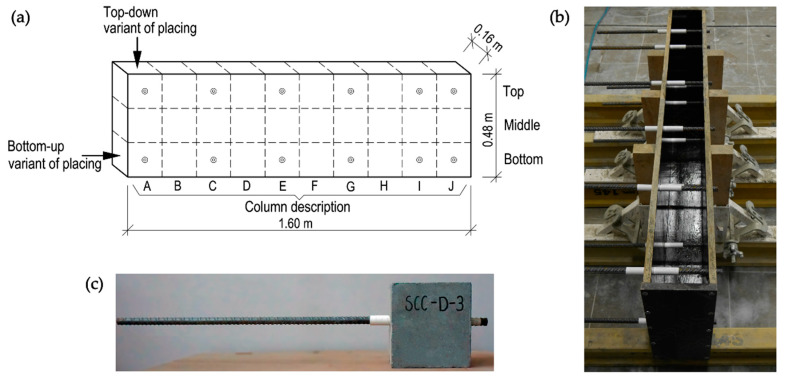
The specimens used in the research: (**a**) schematic view of the element; (**b**) photograph of the formwork used; (**c**) basic model for pull-out test.

For the purpose of this research, elements with dimensions of 160 × 1600 × 480 mm, in which ribbed reinforcing bars were embedded, were made. The element was designed to be divided into cubes of 160 mm side. This is the primary dimension for the basic module for pull-out bond tests involving bars of 16 mm diameter. The dimensions of the basic module—10d × 10d × 10d—were recommended in RILEM TC [28] and EN 10080 [27]. In each basic module, the ribbed reinforcing bar was embedded centrally. The specimens were designed to consist of 12 basic modules for bond tests, the remaining cubes were tested for compressive strength. The reinforcing bars were embedded along the height and length of the element to maintain their perpendicular position to the direction of concreting (Figure 2). For the analysis, the beam was virtually divided into columns marked with the letters A to J starting. A single casting point was established at one edge of the element and was within the area of column A.

The elements were made in two variants of concrete mix placing—traditionally from the top of the form (top-down) and from the bottom (bottom-up). The approach of bottom-up placing of concrete was executed by a specially prepared piping routed above the upper level of the form. Following the principle of connected vessels, the concrete mixture filled the form from below under own weight, initially flowing along the span of the element. When the base is fully covered to the level of the inlet, by lifting the upper poured layer, the mixture spreads evenly along the span. A discharge rate through a funnel was estimated as 0.5 L/s. 

The formwork was stripped after 3 days of concrete curing. The specimens were left in a constant position at laboratory conditions for the entire period of the concrete curing cycle. The concrete was maintained by water sprinkling during this period. The specimens were cut into elementary parts after 21 days, and after 28 days compressive strength and pull-out tests were performed.

### 2.3. Test Procedures

#### 2.3.1. Fresh Mix Properties Tests

Fresh self-compacting mixes were tested in order to evaluate rheological properties as well as air content. The rheological behaviour of the fresh SCC was identified during three tests. A slump flow test that was conducted served as an investigation of both flowability and plastic viscosity [29]. The measured parameters are the final slump flow diameter and the time T_500_ which is related to slump flow time up to a diameter of 500 mm. The passing ability was tested during the L-box test [30]. This property is deduced from the L-box ratio. Finally, the fresh visual segregation index assessed after conducting a slump flow test allowed the segregation resistance to be determined [31]. The rheological tests were performed immediately after the completion of mixing.

Moreover, the percentage content of air bubbles in the mixes was tested with a pressure gauge according to EN 12350-7 [32]. The test was conducted after a temporary stabilization of a mixture in a normative container.

#### 2.3.2. Compressive Tests

The compressive strength was investigated according to EN 12390-3:2009 [33] using cubic elements derived from the test element that was not adopted for the bond test (Figure 2). As the dimensions of the samples were 160 × 160 × 160 mm, it made them designated sizes [34] of samples. It allowed on verify the changes of the compressive strength over the heights and across the lengths of the specimens. Moreover, cubic elements of nominal sizes 150 × 150 × 150 mm were cast from each batch and tested on compressive strength. Overall, 46 cubes were tested in terms of compressive strength.

#### 2.3.3. Bond Strength Tests

There are several different methods of testing a steel-concrete bond. In this research, the pull-out method was adopted. The approach meets the requirements and recommendations of RILEM TC [28] and EN 10080 [27] and is conducted on cubic samples. It is a fundamental method for the assessment of the reinforcing bars–concrete interaction depending on concrete properties or type of reinforcing bars. The method assumption involves the application of a tensile load to the bar anchored in a concrete block. The measured data refers to the load applied to the bar and the relative displacement between steel and concrete.

The main advantage of the method is an assumption of linear deformation along the rebar length; thus the bond stress (τ) is presumed to be constant and could be calculated using Equation (1):

(1) $$\tau =\frac{F}{\pi dl}$$

where *F*, *d* and *l* stand for, respectively, the applied load, reinforcing bar diameter and bond section length. The bond length—*l*—was experimentally adopted as 3.75d. With a greater bond length (the normative value is 5d) the bond forces would be so high that the reinforcing steel would yield and a pull–out failure would not occur. The bond section length was provided by plastic tubes put over the remaining part of the bar.

The pull-out test was performed on 24 basic modules overall. The pull-out load was applied progressively up to the bond failure point. The measurement of the slip of the unloaded end of the rebar was taken using two linear variable displacement transducers (LVDT). A data acquisition system was used.

### 2.4. Result Analysis Procedures

This work analyses three representative bond stresses—the ultimate bond stress (τ_max_) and the critical bond stress (τ_0.25_) and the mean bond stress (τ_m_). For this purpose five bond stresses were retained from each bond-slip curve: τ_0.01_, τ_0.10_, τ_0.25_ and τ_1.00_, in respect to slips s = 0.01 mm, 0.10 mm, 0.25 mm and 1.00 mm, as well as τ_max_, at the time of the bond failure.

The value of the ultimate bond stress τ_max_, which is also known as the bond strength, is proposed in numerous studies [11,12,13,35] given its clear and unambiguous definition. The bond strength corresponds to the bond stress reached at the time of the bond failure. The second representative value found in the literature [36,37] is the critical bond stress corresponding to a slip of the rebar, 0.25 mm, τ_0.25_. As the value of the slip considered in this case (0.25 mm) is always lower than the one obtained when reaching the ultimate bond stress, the value of the critical bond is considered as more conservative and more secure in design than τ_max_. The final value considered here is τ_m_, which is also widely used in the literature [12,35] and is recommended by RILEM TC [28]. It is calculated from Equation (2) as the arithmetic mean of the bond stresses τ_0.01,_ τ_0.10_ and τ_1.00_ corresponding to the slips of 0.01, 0.10 and 1.00 mm, respectively.

(2) $$\tau_{m}=\frac{\tau_{0.01}+\tau_{0.10}+\tau_{1.00}}{3}$$

To exclude the effect of the concrete compressive strength on its bond to reinforcement, the normalization of the representative bond stresses to the square root of the corresponding cube compressive strength of each batch (*τ*/f_cc_^0.5^) was introduced.

## 3. Results

### 3.1. Fresh Mix Properties

The results of rheological properties and air content tests performed on fresh self-compacting mixtures are presented in Table 3. The samples from both mixtures had rheological properties classifying them to the same slump flow (SF2), viscosity (VS1) and passing ability (PL2) classes. The mixtures also had similar stability without any visible signs of segregation or bleeding, which is tantamount to the most favourable value of a fresh visual stability index, namely 0.

The air content of the mixture is generally higher in self-compacting concretes than in normal concrete. It increases with the slump flow and/or the plastic viscosity of the mix and is usually in the range of 2%–5% [38]. Test results at a level of 2% of air content are satisfying.

### 3.2. Compressive Strength

Table 3 presents the mean results of the compressive strength tests carried out on the cubic 150 × 150 × 150 mm reference samples. In turn, the results of the compressive strength tests conducted on the samples cut out of the horizontal elements are given in Table 4.

After the analysis of the compressive strength test results, no definite influence of the direction of concreting on the strength values was found, and any differences remained statistically insignificant. Considering the beam in layers, the strength in the upper layer decreased with the distance from the casting point, and in the bottom layer, the opposite situation occurred. In the middle layer, there was an increase in strength in the middle zone of the element, whereas at both edges it decreased. Given the small number of specimens tested for compressive strength in the bottom and top layers, no particular influence on the direction of placing could be observed. However, it was noted that for the top-down concreting variant, the results scatter was reduced compared to the second variant. The scatter of results in the middle layer was comparable in both variants, and the variation of compressive strength values was alike. Due to relatively minor scatter of results both along the height and length of the elements, it is concluded that any outliers were the outcome of a random strength distribution. The test results correspond to the findings of [39], where also statistically insignificant differences were obtained between the variations in the compressive strength values along the 1.50 m-long deep beams made of HPSCC mixtures.

The mean compressive strength of concrete was 45.64 MPa for the element made from the bottom and 44.91 MPa for the second element. It is worth noting that the compressive strength of the concrete obtained on standard cubic elements was 45.35 MPa and 47.04 MPa for the batches used, respectively, for the bottom-up and top-down placing.

### 3.3. Effect of Placing Direction on Bond Properties

#### 3.3.1. Normalized Bond Strength

Table 5, Table 6 and Table 7 provide the bond test results for the respectively ultimate (τ_max_), critical (τ_0.25_) and mean (τ_m_) normalized bond stresses. It ought to be noted that all the samples indicated the pull-out bond failure mechanism.

Based on the results of pull-out tests, the effect of the mixture placing direction on the normalized bond stresses could be observed, especially in the case of the top bars. While for lower rebars this effect was ambiguous and did not significantly affect either the values of normalized bond stresses or the scatter of the results, for top rebars an increase in the bond stresses was observed for those embedded in the bottom-up concreted elements comparing to top-down variant. For all representative bond stresses, the top bars in the top-down placing variant obtained the lowest values of the normalized bond stresses of the entire scope of tests. The scatter of results observed along the length of the element, in this case, was the highest with a range of 10.8%–21.7% (thus, even 3 times bigger scatter for τ_m_ and τ_0.25_ than for bottom-up concreted elements).

Depending on the considered representative stress, the normalized bond stresses in the top bars of bottom-up casted elements were on average 19.7%, 22.5% and 29.3% higher than the bond stresses in the top bars of top-down casted elements for τ_max_, τ_m_ and τ_0.25_, respectively. In the case of bottom rebars, the normalized bond stress values for elements concreted from the bottom were on average 1.62% lower than the normalized bond stress values for the second concreting variant. One can conclude that this difference was the result of a random scatter of data caused by the small number of samples rather than the influence of the placing direction.

#### 3.3.2. Bond Stress-Slip Relationship

**Figure 3:**
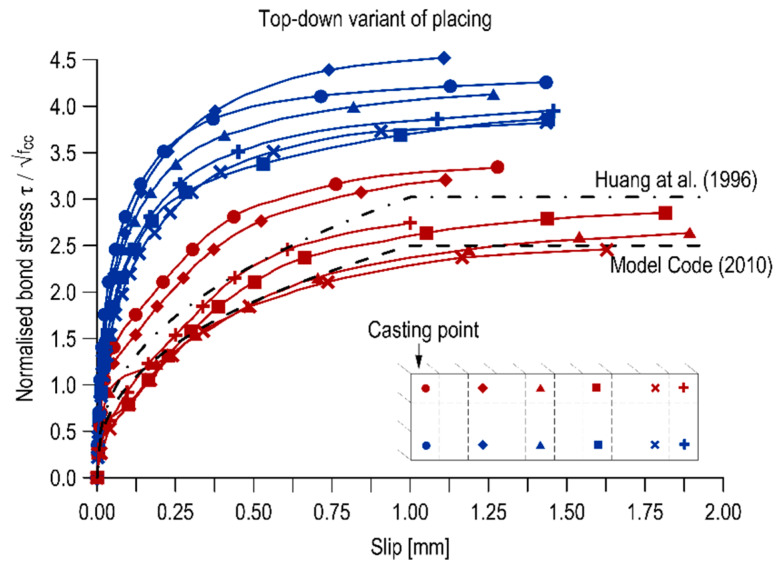
Bond-slip curves in the top-down cast specimen.

**Figure 4:**
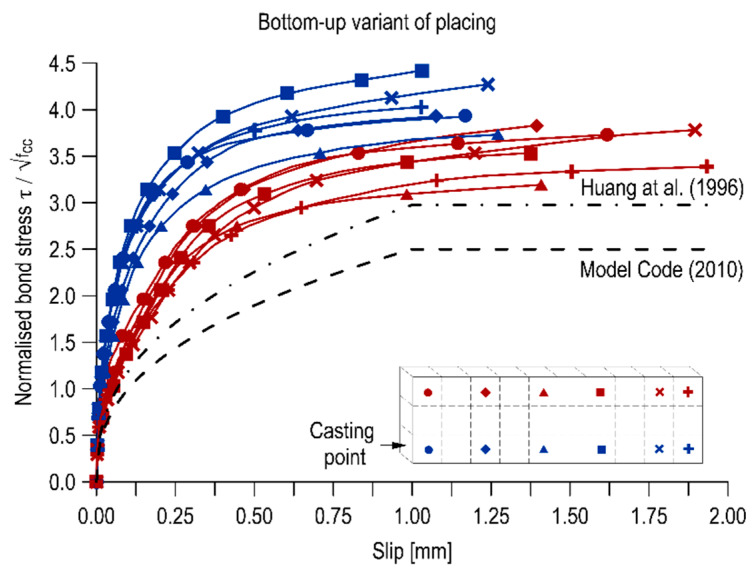
Bond-slip curves in the bottom-up cast specimen.

The relationships between the normalized bond stress and the slip of the bar relative to the concrete are shown in Figure 3 and Figure 4 for the top-down and bottom-up placing variants, respectively. The curves are compared with the bond-slip functions described in the Model Code [40] and Huang et al. [41] regarding normal strength concrete. In both cases, the course, stiffness and shape of the bond stress-slip curves depended on the position of the rebar along the height and length of the elements.

The bond-slip curves for the bottom bars in both versions of the element execution diverged from the standard curves. The top bars in the element concreted from the bottom were also characterized by similar function courses. Most of the top bars of a traditionally constructed element (from the top) exhibited a comparable bond-slip function to that of the Model Code 2010 [40] for good bond conditions and pull-out failure and the function proposed in Huang et al. [41] for good bond conditions in normal strength concrete.

In addition to the comparison of the curves with standard courses, the bond stiffness was analysed. The bond stiffness is a change in bond strains increment relative to bar displacement in concrete. By macroscopic examination of the diagrams, it can be observed that the velocity of increase of the normalized bond stresses is higher for the bottom rebars. For the top rebars, the curves are more flattened, which also translates into generally lower values of the standardized bond stresses. By comparing the two diagrams, it is also concluded that the concreting variant does not affect the bond stiffness of the bottom bars but definitely affects the top ones.

In order to quantify the stiffness parameter of individual curves and verify the conclusions of macroscopic evaluations, an integral of the bond curve up to the slip of 1 mm, corresponding to bond stress τ_1.00_, was calculated using Equation (3), which is referred to as τ_stiff_.

(3) $$\tau_{stiff}=\int_{0}^{s_{1.00}}\tau_{norm}ds/s_{1.00}$$

where τ_norm_ is the normalized representative bond stress and s_1.00_ corresponds to a slip of 1 mm. In Table 8, the calculated normalized bond stress values from the definite integrals are presented.

The distribution of the calculated values overlaps with other representative values of normalized bond stresses. The integral values for rebars in the bottom areas of the elements are close to each other and are on average 3.46 for the top-down variant and 3.44 for the bottom-up variant. However, there is a significant decrease in these values for the top rebars, which is additionally considerably higher for the top-down placing variant. In the case of traditional concreting, the normalized bond stress of the top bar against the bottom bar decreases on average by 40.1% and in the case of bottom-up concreting by an average of 21.4%. The higher bond stiffness is associated with a better concrete quality under the ribs of the rebar, so that the pull-out force, necessary to move a section of the bar, must be increased.

#### 3.3.3. Change of Bond Stresses over Height

**Figure 5:**
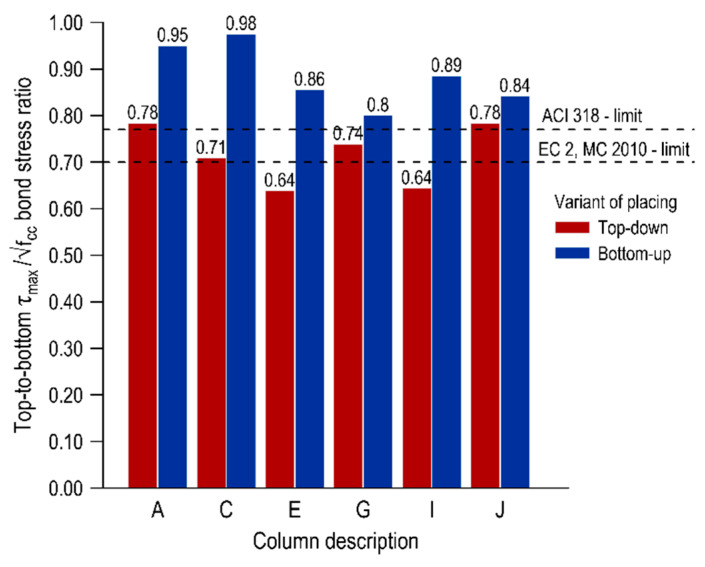
Top-to-bottom ratio for normalized ultimate bond stresses.

**Figure 6:**
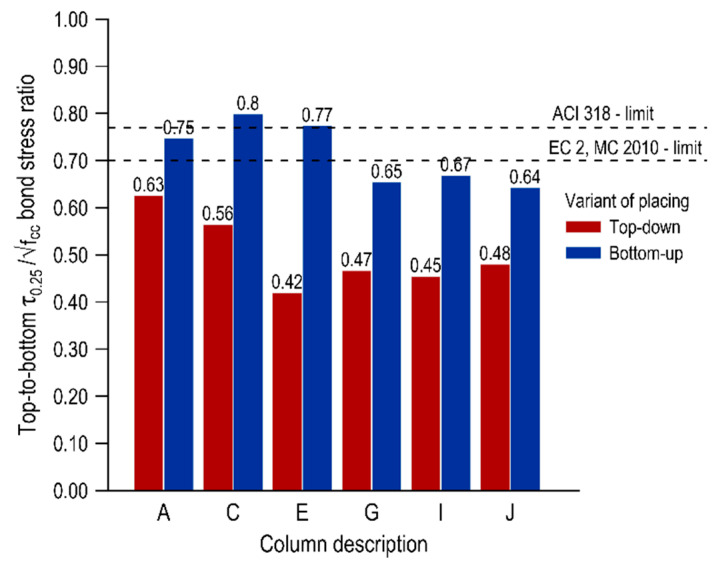
Top-to-bottom ratio for normalized critical bond stresses.

**Figure 7:**
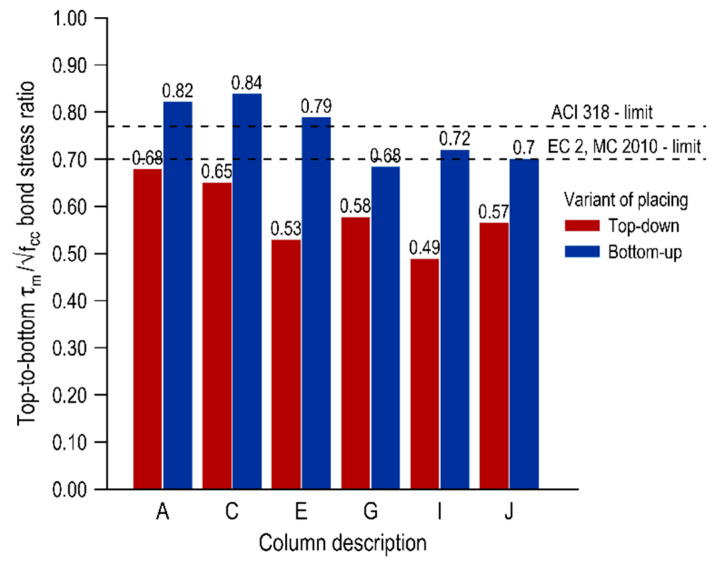
Top-to-bottom ratio for normalized mean bond stresses.

A deterioration of the bond conditions in the top layer of the elements can be seen in the whole scope of the research, and the level of decrease is dependent on the variant of mixture placing and the distance from the casting point. The top-bar effect can be quantified by comparing the normalized bond stresses of the bars in the top parts of the element to the bond stresses of the bottom bars. Figure 5, Figure 6 and Figure 7 present the top-to-bottom ratios for individual representative normalized bond stresses (τ_max_, τ_0.25_, τ_m_, respectively) with the distinction of both mix placing variants. Additionally, the limits for good bond conditions specified in international standards are marked in the graphs-for the American standard ACI 318 [8] which is 0.77 and for European standards [10,40] which is 0.7.

In terms of the ultimate bond stress, the top-to-bottom ratios for the element cast from the top are in the range of 0.64–0.78 and for the element made in the second variant in the range of 0.80–0.98. These results are in line with those in the studies [12]. In the case of a bottom-up variant, all values are above the standard levels of good bond conditions, and in the area closest to the casting point (columns A and C) the normalized bond stresses of the bottom and top bars do not differ significantly. In the traditional concreting variant, much greater discrepancies in the bond conditions along the element are observed, and these results remain mostly above the European limit (except for columns E and I). The results for mean and critical normalized bond stresses appear to be slightly different, and the top-to-bottom ratios in the top-down casted element are below the EC 2 limit with τ_m_ between 0.44–0.65 and τ_0.25_ 0.42–0.63. These values are similar to those obtained in conventional concretes. In the case of an element concreted from the bottom, the values of top-to-bottom ratios depend on the distance from the casting point. Overall, the ratios for the τ_0.25_ and τ_m_ are in the range of 0.64–0.80 and 0.66–0.80, respectively. The values in columns A–E are above the European limits and subsequently, the values decrease. It may be noted that the scatter of the results along the span is smaller in the case of a bottom-up casted element.

The reduced values of the top-to-bottom ratios for the more conservative representative normalized bond stresses (τ_0.25_ and τ_m_) were associated with the bond stiffness variation for bars at different heights, which was further described and explained in Section 3.3.2. In general, the bottom bars achieved a higher level of bond stress with smaller slip values than the top bars, even though the final values of the ultimate bond stress were comparable to each other.

Throughout the entire scope of the tests, it is noted that placing concrete from the bottom has a positive effect on the uniformity of bond quality over the height of the elements. Such an improvement is explained by the enhancement of the contact surface quality in the upper part of the elements. Given the fact that the mixture placed from the bottom is successively lifted in the form together with its deaeration, fewer air bubbles are accumulated under the rebar, thereby improving the interaction of the bar with the surrounding concrete. This translates into both the bond stress values of the top bars and the stiffness of bond-slip curves.

#### 3.3.4. Change of Bond Stresses over the Length

**Figure 8:**
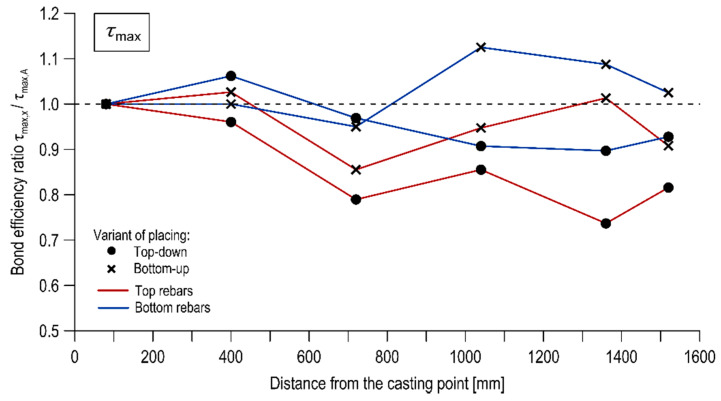
Bond efficiency ratio as a function of rebar position for the ultimate bond stress.

**Figure 9:**
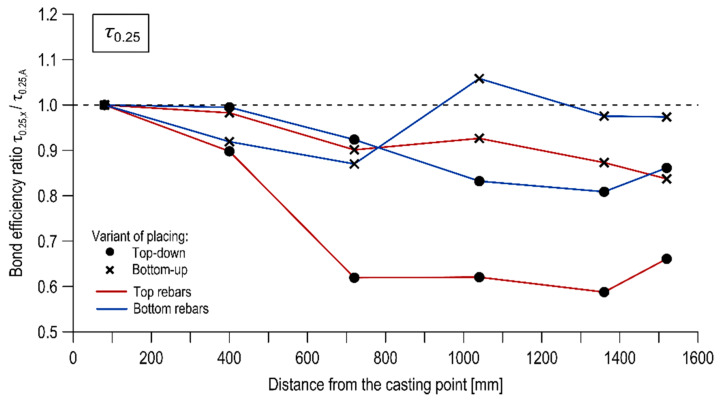
Bond efficiency ratio as a function of rebar position for the critical bond stress.

**Figure 10:**
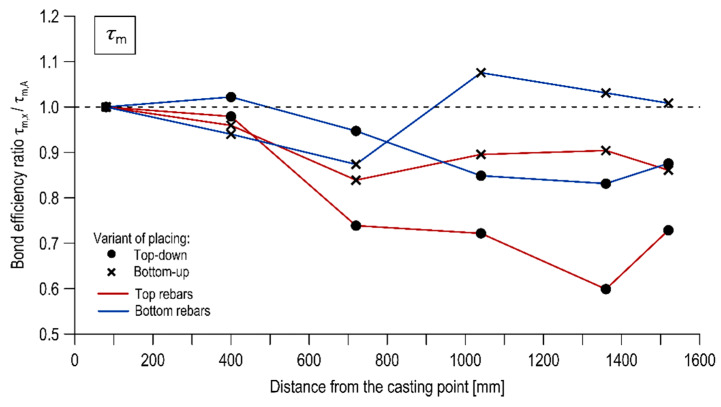
Bond efficiency ratio as a function of rebar position for the mean bond stress.

The effect of the rebar’s distance from the casting point on the bond was examined by comparing the normalized ultimate, critical, and mean bond stresses between the successive reinforcement bars across the length of the specimens. The test results in this respect are presented in Table 5, Table 6 and Table 7. Figure 8, Figure 9 and Figure 10 illustrate the course of change of the bond efficiency ratio along the element defined as the ratio of the normalized bond stress of the considered rebar to the rebar located at the casting point (specimen of column A), respectively for τ_max_, τ_0.25_, τ_m_.

The studies revealed the presence of the effect of the rebar’s distance from the casting point. The examined effect strongly depended on the direction of concrete mix placing. In the case of concrete placing from the top, it may be stated that the bond between the rebar and the concrete is decreasing as the distance between the rebar and the point of concrete placing is increasing. This tendency is particularly noticeable for bars located in the upper part of the specimens. The reduction of the representative normalized bond stress between the top bars of column A and column J was 18.6%, 33.6%, 27.2% respectively for τ_max_, τ_0.25_, τ_m_. The following decrease was noted for the bottom bars: 7.3%, 13.8%, 12.2%. The change of bond stresses over length was significantly reduced when the concrete mix was placed from the bottom of the form. The drop in bond strengths of the top bars between column A and column J was 9.1%, 16.6%, 13.8% for τ_max_, τ_0.25_, τ_m_ respectively, whereas no significant difference in bond strength values was noted for the bottom rebars.

**Figure 11:**
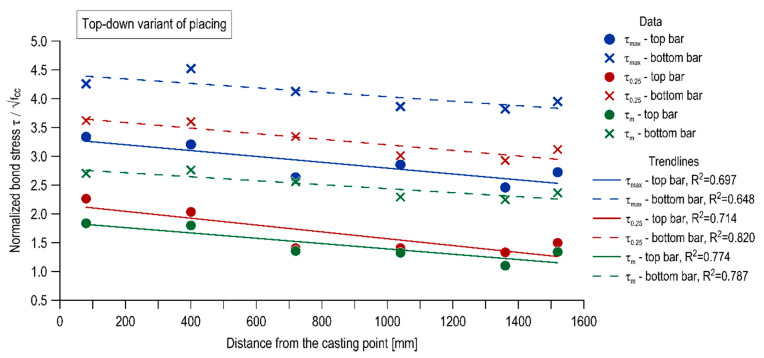
Effect of distance from casting point in the top-down variant of placing.

**Figure 12:**
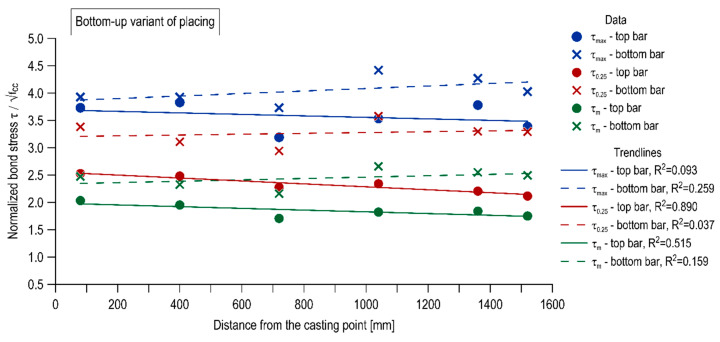
Effect of distance from casting point in the bottom-up variant of placing.

The influence of the mixture placing direction on the effect of distance from casting point is shown in Figure 11 and Figure 12 in the form of inserted linear trendlines, respectively for the top-down variant of placing and the bottom-up one. It is observed that for the specimen cast traditionally, the linear regression led to a strong linear relation (R^2^ = 0.648–0.82), with a clearly negative slope. Thus, the bond strength had a regressive nature with distance from the casting point. Alternatively, for the bottom-up casted specimen, the linear regression led to generally weak (with one exception for the top bar stress of τ_0.25_) linear relation (R^2^ = 0.037–0.89) and the slope is approximately a constant function.

The bottom-up placing method allows continuous self-venting and self-compacting of layers during concreting. This results in a more homogeneous distribution of representative bond stresses along the element compared to traditional placing from the top.

## 4. Discussion

### 4.1. Evaluation of Casting Direction

The casting technology from the bottom of the form results in initial mixture flowing along the element until the height of the outlet, through which it is injected, is reached. Then, the previously introduced mixture is gradually lifted upwards and distributed along the element. The lifted concrete layer is continuously vented and self-compacted during casting works.

The analysis of the bond-slip function course, the values of bond stress along the height as well as along the span indicated a beneficial effect of the bottom-up placing method on the phenomenon of the bond between reinforcing steel and concrete. As the main explanation for the favourable result of the mixture application from the bottom of the form, one should mention the opportunity of its more effective self-deaeration and self-compaction. This reduced the number of air bubbles in the rebar-concrete contact zone and decreased the settlement of the mixture under the upper rebar. The concrete cover under the bars in the upper part of the element created in this way was of better quality than in the case of traditional casting from the top of the form. An additional justification for the selected factors influencing the improvement of the bond of the upper bars is the lack of significant influence of the concrete casting direction on the bond of the lower bars.

### 4.2. Comparision to HPSCC

When comparing the results of tests on SCC concrete presented in this article with the findings obtained in identical tests on HPSCC concrete [26], observations can be made on a slightly wider spectrum of cases. The concrete placing from the bottom of the form both for SCC and HPSCC generally led to the improvement of bond characteristics (bond stiffness, bond strength, top-bar effect, the effect of the rebar distance from the casting point). This was associated with effective self-deaeration and self-compaction of the mixtures, which manifested itself in a reduction of their settlement. This observation was independent of the composition of self-compacting mixtures and/or proportions of their ingredients as well as the strength of the hardened concrete. Therefore, it can be assumed that the beneficial effect of bottom-up placing on bond characteristics is related to the overall rheological properties of SCC mixtures. Hence, the results presented are promising and encourage more comprehensive research on this issue.

### 4.3. Future Research

New-generation concretes, in general, demonstrate improved bond properties in comparison to traditional concrete. The main factor contributing to enhanced bond conditions is the more sealed structure of self-compacting concretes and high-performance self-compacting concretes than normal concrete thanks to the use of mineral additives and microfillers. The studies which have been carried out up to this moment [2,3,4,26] demonstrate that the microstructure of concretes can be significantly tightened by their more effective deaeration during the concrete placing from below. The improvement of the bond phenomenon within an entire element, namely the elimination or significant reduction of the zone of poor bond conditions, raises doubts regarding the very strict design recommendations in the case of the anchorage length of reinforcing bars. The current design guidelines [8,9,10,40] are based on tests of normal-strength traditional concretes and do not take into account the different behaviour of new-generation concretes and alternative concreting technologies. Reducing the required anchorage length and lap length of rebars would allow steel consumption to be reduced and hence potential cost savings of the reinforced concrete element. Such technical solutions and reduction techniques are compatible with the commonly adopted trend of sustainable construction.

Nevertheless, the scope of the current studies is insufficient to draw definitive and extensive conclusions, only preliminary ones. Further research on the possibility of improving the bond and strength properties of self-compacting concretes has a positive impact on public opinion towards these concretes in the construction industry. The authors suggest that the investigations should be extended to different self-compacting mixtures, different placing velocities, other structural elements (columns, walls) in order to properly verify the effect of bottom-up placing on bond properties.

## 5. Conclusions

The research enables preliminary conclusions to be drawn concerning the influence of the SCC mixture placing from the bottom of the form on the strength characteristics of the hardened concrete, as well as on the quality of steel-concrete bond conditions. The results analysis of the conducted tests indicates that:

- There is no significant influence of the direction of the concrete mix placing on the concrete compressive strength in the tested element.
- For the bars located in the bottom part of the tested element, the direction of concreting had no significant effect on the bond stiffness. In contrast, a significant improvement in the bond stiffness was observed for rebars positioned in the top part of the specimen when the mixture was placed from the bottom of the form.
- Improved reinforcing steel-to-concrete bond when placing concrete bottom-up is a result of a limitation of negative phenomena, such as top-bar effect and the effect of distance from the casting point.

The concrete technology from the bottom of the form caused a reduction in the amount of air bubbles in the rebar-concrete contact layer and a reduction in the settlement of the mixture under the top rebar. The generated concrete cover under the rebars was characterized by better quality when compared to the traditional concreting from the top of the form. Therefore, the bottom-up placing contributed to the uniformity of the bond conditions’ quality in the test element and an overall improvement of the bond properties, especially in its top part. Corresponding conclusions of the positive impact of the bottom-up placing method on bond properties were drawn from the authors’ research on HPSCC concretes. Given the increasing implementation of the bottom-up placing technology of SCC in practice and the lack of any normative references, a more comprehensive study should be conducted in a wider spectrum of cases.

## Figures and Tables

**Table 1:** Composition of self-compacting concrete (SCC) mix.

Composition [kg/m^3^]	SCC–FA20
Cement CEM II/B-V 32.5R	360
Water	160
Sand 0–2 mm	700
Gravel aggregate 2–8 mm	350
Gravel aggregate 8–16 mm	350
Fly ash	90
Superplasticizer	3.0
Binder content	450
Water/binder ratio	0.36
Fly ash level	20%

**Table 2:** Mechanical parameters of reinforcing bars (B500SP).

Parameter	Symbol	Units	Producer Limits	Measured Values	
				Average	St. deviation
Yield strength	f_y_	N/mm^2^	≥500	558.1	6.8
Tensile strength	f_t_	N/mm^2^	≥575	653.2	11.4
Ratio	f_t_/f_y_	—	1.15 ÷ 1.35	1.17	0.01
Uniform elongation	ε_u,k_	%	≥8.0	13.54	0.91

**Table 3:** Fresh properties and cubic compressive strength test results.

Mix Batch	Slump Flow [mm]	Slump Flow Class	Slump Flow Time T_50_ [s]	Viscosity Class	L-Box Ratio	L-Box Class	Fresh Visual Stability Index	Air Content [%]	Compressive Strength f_cc_ [MPa]
SCC-FA20-I	705	SF2	1.5	VS1	0.89	PL2	0	1.9	47.04
SCC-FA20-II	720	SF2	2	VS1	0.90	PL2	0	2.0	45.35

**Table 4:** Compressive strength test results for horizontal elements.

Casting Direction	Layer	Compressive Strength f_cc,i_ [MPa]											
		Column										Mean	Cov
		A	B	C	D	E	F	G	H	I	J		
Top-down	Top	*	46.2	*	44.9	*	43.9	*	42.9	*	*	44.47	2.8%
	Middle	39.9	42.5	47.9	48.0	45.2	49.8	42.5	47.5	44.8	42.9	45.11	6.6%
	Bottom	*	44.4	*	44.7	*	45.1	*	46.5	*	*	45.15	1.8%
Bottom-up	Top	*	46.6	*	42.6	*	43.9	*	47.4	*	*	45.12	4.3%
	Middle	39.2	45.1	45.6	45.4	48.7	46.0	47.5	48.7	47.4	43.0	45.66	6.0%
	Bottom	*	42.3	*	45.3	*	46.9	*	49.9	*	*	46.08	6.0%

* sample used for pull-out test.

**Table 5:** Normalized ultimate bond stress for the rebars in all elements.

Casting Direction	Layer	Normalized Ultimate Bond Stress τ_max_/√f_cc_											
		Column										Mean	Cov
		A	B	C	D	E	F	G	H	I	J		
Top-down	Top	3.34	*	3.20	*	2.63	*	2.85	*	2.46	2.72	2.87	10.8%
	Bottom	4.26	*	4.52	*	4.13	*	3.86	*	3.82	3.95	4.09	6.0%
Bottom-up	Top	3.73	*	3.83	*	3.19	*	3.53	*	3.78	3.39	3.57	6.4%
	Bottom	3.93	*	3.93	*	3.73	*	4.42	*	4,27	4,02	4.05	5.7%

* sample used for compressive strength test.

**Table 6:** Normalized critical bond stress for the rebars in all elements.

Casting Direction	Layer	Normalized Critical Bond Stress τ_0.25_/√f_cc_											
		Column										Mean	Cov
		A	B	C	D	E	F	G	H	I	J		
Top-down	Top	2.26	*	2.03	*	1.40	*	1.40	*	1.33	1.50	1.66	21.7%
	Bottom	3.62	*	3.60	*	3.34	*	3.01	*	2.93	3.12	3.27	8.3%
Bottom-up	Top	2.53	*	2.48	*	2.28	*	2.34	*	2.20	2.11	2.32	6.2%
	Bottom	3.38	*	3.11	*	2.94	*	3.58	*	3.30	3.29	3.27	6.2%

* sample used for compressive strength test.

**Table 7:** Normalized mean bond stress for the rebars in all elements.

Casting Direction	Layer	Normalized Mean Bond Stress τ_m_/√f_cc_											
		Column										Mean	Cov
		A	B	C	D	E	F	G	H	I	J		
Top-down	Top	1.84	*	1.80	*	1.36	*	1.33	*	1.10	1.34	1.46	18.3%
	Bottom	2.70	*	2.76	*	2.56	*	2.29	*	2.25	2.37	2.49	8.0%
Bottom-up	Top	2.03	*	1.95	*	1.71	*	1.82	*	1.84	1.75	1.85	6.1%
	Bottom	2.47	*	2.32	*	2.16	*	2.66	*	2.55	2.49	2.44	6.6%

* sample used for compressive strength test.

**Table 8:** Bond stiffness parameter for the whole scope of research.

Casting Direction	Layer	Bond Stiffness Parameter τ_stiff_											
		Column										Mean	Cov
		A	B	C	D	E	F	G	H	I	J		
Top-down	Top	2.64	*	2.48	*	1.77	*	1.88	*	1.67	2.02	2.08	17.39%
	Bottom	3.73	*	3.85	*	3.53	*	3.15	*	3.17	3.31	3.46	7.81%
Bottom-up	Top	2.91	*	2.87	*	2.54	*	2.72	*	2.65	2.51	2.70	5.59%
	Bottom	3.43	*	3.35	*	3.10	*	3.75	*	3.54	3.49	3.44	5.76%

* sample used for compressive strength test.
